# Beidseitiges Capsular-Block-Syndrom 7 Jahre nach Kataraktoperation

**DOI:** 10.1007/s00347-021-01335-2

**Published:** 2021-02-18

**Authors:** Tyll Jandewerth, Thomas Kohnen

**Affiliations:** Klinik für Augenheilkunde, Goethe-Universität Frankfurt am Main, Theodor-Stern-Kai 7, 60590 Frankfurt am Main, Deutschland

## Anamnese

Eine 48-jährige Patientin arabischer Herkunft stellte sich mit einer seit 1 Tag bestehenden, schmerzlosen Visusminderung am linken Auge nachts im Notdienst vor. Sie beschreibt ein verschwommenes Sehen wie durch einen Nebel. Sie könne jedoch nicht sicher sagen, ob die Visusminderung nicht auch schon vorher bestanden hätte. Am rechten Auge würden keine Beschwerden auftreten. An Voroperationen habe die Patientin vor 7 Jahren eine Kataraktoperation mit Implantation einer Intraokularlinse (IOL) extern an beiden Augen durchführen lassen. Sonst sei keine weitere Augenerkrankung oder -behandlung bekannt. Allgemeinerkrankungen würden nicht bestehen, ebenso keine regelmäßige Medikamenteneinnahme und keine bekannten Allergien.

## Klinischer Befund

**Figure 1:**
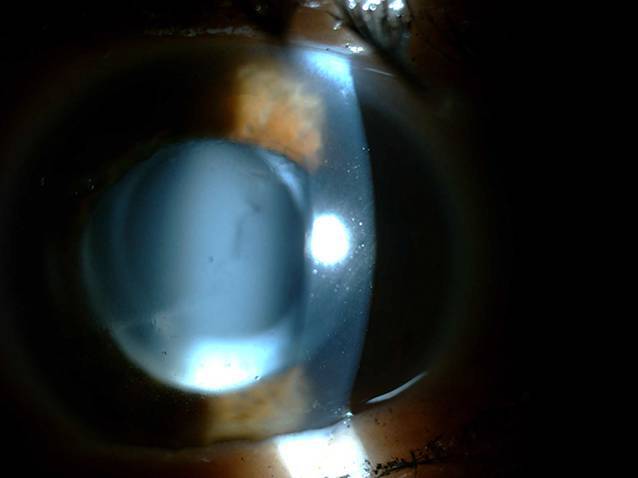

Die klinische Untersuchung ergab beidseits eine geringe Hyperopie sowie einen Astigmatismus (rechtes Auge: +1,0/−0,50/97°, linkes Auge: +1,25/−1,25/24°). Die Sehschärfe betrug mit Korrektur auf dem rechten Auge 0,6, auf dem linken Auge 0,4. Der Augendruck lag mit 12 mm Hg rechts bzw. 10 mm Hg links jeweils im Normbereich. Die vorderen Augenabschnitte waren unauffällig, es zeigte sich eine regelrechte Pseudophakie mit lediglich einer weißlichen Trübung mehr links- als rechtsseitig, welche bei der Erstuntersuchung hinter der einliegenden IOL lokalisiert wurde (Abb. 1).

Bei der Untersuchung des Augenhintergrundes in Mydriasis zeigte sich beidseits ein unauffälliger und regelrechter Befund bei jedoch reduziertem Einblick linksseitig.

Die Motilität war in allen diagnostischen Blickrichtungen frei und die Reagibilität der isokoren Pupillen regelrecht.

## Weiteres Procedere

Die Patientin wurde bei Verdacht auf das Vorliegen eines Nachstars über eine Neodym-dotierter Yttrium-Aluminium-Granat-Laser-Kapsulotomie (YAG-Kaspulotomie) aufgeklärt und für den nächsten Tag erneut einbestellt. Die Durchführung der YAG-Kapsulotomie am linken Auge zeigte sich jedoch deutlich erschwert, eine Fokussierung der Laserbündel war nicht sicher möglich. Nach mehreren erfolglosen Versuchen wurde die Verdachtsdiagnose einer Materialermüdung des einliegenden Linsenmaterials gestellt. Die Patientin erhielt einen neuen Termin in der Spezialsprechstunde für Kunstlinsen. Bei Vorstellung zeigte sich linksseitig weiterhin eine reizfreie Pseudophakie. Hier wurde nun bei genauer Untersuchung eine gut zentrierte, klare Hinterkammerlinse festgestellt. Dahinter zeigte sich ein mit weißlich-trübem Material aufgedehnter, gespannter Raum zwischen Rückseite der Hinterkammerlinse und hinterer Linsenkapsel. Es konnte nun die endgültige Diagnose eines Capsular-Block-Syndroms (CBS) gestellt werden.

## Hintergrundinformationen

Es handelt sich bei der vorgestellten Kasuistik um eine seltene Komplikation nach einer Kataraktoperation mit einer Inzidenz von unter 1 % [12]. Ursache eines Capsular-Block-Syndroms ist eine Flüssigkeits- oder Gewebeansammlung zwischen der IOL und der hinteren Linsenkapsel. Hierdurch kommt es über einen variablen Zeitraum zu einer Aufdehnung und Eintrübung des hinteren Kapselsacks.

Neben der Kataraktoperation bei altersbedingter Linsentrübung kann als Risikofaktor für die Entstehung eines Capsular-Block-Syndroms nach IOL-Implantation auch eine sekundäre oder kongenitale Katarakt angedacht werden.

Eine kongenitale Katarakt halten wir in unserem Fall aber für unwahrscheinlich, da anamnestisch bis auf die Phakoemulsifikation mit Implantation einer Hinterkammerlinse vor 7 Jahren (Alter des Patienten damals: 41 Jahre) kein ophthalmochirurgischer Eingriff vorgenommen worden war. Bei einer kongenitalen Katarakt wäre sehr wahrscheinlich bereits in der frühkindlichen Entwicklung eine operative Behandlung durchgeführt worden, um die Gefahr der Entstehung einer Amblyopie zu reduzieren. Auch spricht der beidseitige Visusanstieg auf 1,0 nach erfolgter operativer Behandlung des Capsular-Block-Syndroms bei sonst blandem Befund eher gegen eine kongenitale Katarakt, da hier bei ausgebliebener operativer Behandlung in der frühkindlichen Phase von einer amblyopiebedingten Visusminderung auszugehen ist.

Zusätzlich halten wir eine sekundäre Katarakt bei blander Allgemein- und Medikamentenanamnese ebenso für unwahrscheinlich.

Das vorgestellte Krankheitsbild kann anhand seines zeitlichen Auftretens in 3 verschiedene Formen unterteilt werden: intraoperativ, früh-postoperativ und spät-postoperativ [1].

Das intraoperative CBS entsteht bei ausgeprägtem hinterem Polstar, da für die Hydrodissektion größere Flüssigkeitsmengen oder ein höherer Injektionsdruck vonnöten sind [1]. Hierbei kann es zu einer Verlagerung der Linse nach anterior mit Flüssigkeitsansammlung und intrakapsulärem Druckanstieg kommen. Die Gefahr der Kapselruptur ist in diesem Fall eine häufige Komplikation [2].

Das früh-postoperative CBS entsteht innerhalb der ersten 2 Wochen postoperativ. Ursächlich sind hier verschiedene Mechanismen. Eine Ursache kann im Kapselsack hinter der IOL verbliebene viskoelastische Substanz (OVD) sein [2]. Weiterhin können im Kapselsack verbliebene Reste von OVD oder Linsen‑/Kapselresten einen osmotischen Gradienten erzeugen [4, 5]. Folglich kommt es zu einem Flüssigkeitseinstrom und einer Akkumulation von Flüssigkeit hinter der Hinterkammerlinse. Allen Mechanismen gleich ist die mögliche Verlagerung der IOL nach anterior mit Abnahme der Vorderkammertiefe. Die Patienten bemerken, neben der verschwommenen Sicht durch die in den Kapselsack einströmende Flüssigkeit, eine zunehmende Kurzsichtigkeit, welche sich auch in der Refraktion widerspiegelt. Dies wird auch als „myopic shift“ bezeichnet [3, 4].

Bei der spät-postoperativen Form kommt es zu einer Proliferation der an der Hinterkapsel verbliebenen Linsenepithelzellen. Eifrig [7] konnte zeigen, dass diese Kollagen und weitere extrazelluläre Matrix bilden, sodass auch bei dieser Form im Laufe der Zeit Flüssigkeit in den hinteren Kapselsack eintreten kann. Es bildet sich eine weißliche Trübung, weshalb diese Form des CBS auch als „Lacteocrumenasia“ bezeichnet wird. Im Mittel tritt die spät-postoperative Form des CBS 3,8 Jahre nach einer Kataraktoperation auf [6].

Zusätzlich zu dem zeitlichen Auftreten des CBS kann eine Einteilung in eine fibrotische, eine inflammatorische und eine nichtproliferative Form erfolgen. Die fibrotische Form tritt eher in einem langen Zeitraum postoperativ auf und gehört somit zum spät-postoperativen CBS [3]. Zeigen sich Entzündungszeichen in der Vorderkammer oder im hinteren Kapselsack, kann zusätzlich die Diagnose eines inflammatorischen CBS gestellt werden [1]. Dieses tritt innerhalb weniger Tage bis Wochen postoperativ auf und wird somit häufig der früh-postoperativen Form zugerechnet. Bei der nichtproliferativen Form verbleibt beispielsweise etwas OVD im hinteren Kapselsack, sodass hierdurch innerhalb weniger Tage postoperativ ein erweiterter hinterer Kapselsack durch osmotisch bedingten Flüssigkeitseinstrom auftritt. Diese Form wird auch mit dem früh-postoperativen CBS assoziiert [1] und kann, wie im Falle unserer Patientin, auch noch Jahre nach der Implantation der Hinterkammerlinse auftreten.

Die Diagnose eines CBS kann primär klinisch gestellt werden, wenn in der Spaltlampenmikroskopie ein dilatierter hinterer Kapselsack und eine verringerte Vorderkammertiefe dargestellt werden. Unterstützend kann die postoperative Myopisierung („myopic shift“) hinzugezogen werden. Zusätzlich können ein Vorderabschnitts-OCT, eine Scheimpflug-Aufnahme (z. B. Pentacam; Oculus Optikgeräte GmbH, Wetzlar, Deutschland) und eine Ultraschallbiomikroskopie durchgeführt werden, um die Diagnose zu sichern [1]. Bei diesen bildgebenden Untersuchungen sind die Verlagerung der IOL nach anterior sowie der erweiterte hintere Kapselraum besser darstellbar.

Therapeutisches Ziel ist es, den erweiterten hinteren Kapselsack zu entlasten und somit die Adhäsion des vorderen Kapselrandes mit der einliegenden Kunstlinse aufzuheben. Jedoch sollte jede Therapieoption individuell abgewogen werden, da gelegentlich auch ein abwartendes Verhalten mit spontaner Besserung der Beschwerden auftreten kann. Neben der Gabe von topischen Steroiden beim inflammatorischen CBS [3] existieren weitere interventionelle Optionen.

Einfach durchführbar ist eine YAG-Kapsulotomie der hinteren Kapsel. Hierbei kommt es zu einer Entleerung der im hinteren Kapselsack gelegenen Flüssigkeit in den Glaskörperraum. Direkt nach Entleerung können eine Reposition der nach anterior verlagerten IOL und eine Abflachung des hinteren Kapselraumes beobachtet werden [8]. Dieses Verfahren sollte jedoch keinesfalls beim Vorliegen von Entzündungszeichen mit Verdacht auf ein inflammatorisches CBS durchgeführt werden, da hierbei die Gefahr einer Verschleppung der Entzündung in den Glaskörperraum und das Auftreten einer Endophthalmitis vorliegt [11]. Insgesamt kann die hintere YAG-Kapsulotomie auch nur erschwert bei der postoperativen Form durchgeführt werden, da die weißliche trübe Flüssigkeit hinter der IOL eine Fokussierung deutlich erschwert. Hier kann eine YAG-Kapsulotomie der vorderen Kapsel mit kombinierter peripherer YAG-Iridotomie versucht werden [10].

Bei der operativen Versorgung eines CBS haben Qu et al. mit folgender Technik gute Ergebnisse erzielt [9]: Mittels einer eingeführten 27-G-Nadel wurde Druck auf die IOL ausgeübt und diese mehrmals nach posterior geschoben, sodass es zu einer Entleerung der Flüssigkeitsansammlung in die Vorderkammer kam. Zusätzlich wurde der hintere Kapselsack ausgiebig gespült, und es wurden evtl. Adhäsionen der vorderen Kapsel mit der einliegenden IOL entfernt.

## Therapie

Unsere Patientin erhielt eine operative Behandlung des CBS am stärker betroffenen linken Auge, im Verlauf wurde die gleiche Operation auch auf dem rechten Auge durchgeführt.

Nach Anlegen von 2 Parazentesen erfolgte das Eingehen mit einem Spatel hinter die IOL, hierbei kam es schon zu einer deutlichen Entlastung des posterioren Flüssigkeitsdepots. Nach dem leichten Dislozieren der Kunstlinse konnten das gesamte Flüssigkeitsdepot im hinteren Kapselraum sowie zusätzlich der vorliegende Nachstar chirurgisch entfernt werden, eine hintere Kapsulotomie wurde nicht durchgeführt. Die IOL wurde wieder repositioniert, anschließend die Pupille verengt, und die Parazentesen wurden mittels Hydroinsufflation verschlossen. Abschließend erfolgte die Anlage eines Augensalbenverbandes mit antibiotischer Augensalbe.

## Verlauf

Die initial gestellte Verdachtsdiagnose eines Nachstars bestätigte sich nicht. Dies lag zum einen am gescheiterten Versuch, während der YAG-Kapsulotomie die Laserstrahlenbündel zu fokussieren, sowie zum anderen an der mangelnden Erfahrung des Erstuntersuchers. Eine Materialermüdung mit Eintrübung des einliegenden Linsenmaterials stellte in diesem Fall eine weitere mögliche Diagnose dar. Durch genaue Spaltlampenmikroskopie hätte jedoch bereits bei der Erstvorstellung die weißliche Flüssigkeitsansammlung im erweiterten hinteren Kapselsack bemerkt und die Verdachtsdiagnose eines CBS gestellt werden können.

Zur Zweitvorstellung der Patientin wurde nun in der Spaltlampenmikroskopie ein links- mehr als rechtsseitiges Capsular-Block-Syndrom diagnostiziert. Bei der hinter der IOL befindlichen Flüssigkeit konnte eine entzündliche Komponente nicht sicher ausgeschlossen werden. Die Durchführung einer YAG-Kapsulotomie der hinteren Kapsel hätte zu einer Entleerung des unbekannten Materials in den Glaskörperraum geführt und somit zu einer möglichen schweren intraokularen Entzündung. Wir wählten deshalb die operative Versorgung beider Augen. Hierdurch zeigte sich bei der Patientin ein beidseitiger Visusanstieg auf bestkorrigiert 1,0. Auch hierbei ist natürlich eine schwere intraokulare Entzündung eine mögliche Folgekomplikation. Jedoch ist das Risiko nach Absaugen des möglichen inflammatorischen Materials deutlich geringer.

## Fazit für die Praxis

- Bei trüb erscheinender Hinterkammerlinse mit der Verdachtsdiagnose Nachstar bzw. Materialermüdung ist eine genaue und ausführliche Spaltlampenmikroskopie sinnvoll und oftmals ausreichend für die Diagnosestellung eines Capsular-Block-Syndroms.
- Die verschiedenen Formen des Capsular-Block-Syndroms mit ihrem zeitlichen Auftreten sollten v. a. hinsichtlich der Therapieentscheidung berücksichtigt werden.
- Die Behandlung eines Capsular-Block-Syndroms mittels YAG-Kapsulotomie der hinteren Linsenkapsel sollte bei unklaren intraokularen Entzündungszeichen zurückhaltend gestellt werden. Hier ist die operative Absaugung des intrakapsulären Flüssigkeitsdepots eine deutlich risikoärmere Methode.
